# Atlanta Academy of Medicine

**Published:** 1875-05

**Authors:** 


### Atlanta Academy of Medicine.

ROBERT BATTEY, M.D., Reporter.

Atlanta, Ga., 9th March, 1875.

Dr. A. A. Woodhull, in the absence of the President and Vice-President, was called to the chair.

Dr. J. G. Westmoreland desired to call the attention of members to an important means for the relief of obstructions in the bowels. He had been in the habit for twenty years of throwing into the intestines a large quantity of warm water, for obstruction supposed to be intussusception, impacted cæcum, etc., with decided success. He had used the ordinary syringe for this purpose; more recently the cylinder pump of considerable power, by which he had relieved perfectly a case in extremis by a force that might be considered *risky.* He now uses the rubber-bulb syringe, which has sufficient power for all purposes. From one to two or more gallons of water may thus be introduced through the rectum, washing out impaction and expanding so as to draw out intussuscepted portions, knots, etc., that may lead to the obstruction. With the view of testing this plan for the reduction of strangulated hernia, as suggested by Dr. Battey last year, he had tried it partially with a case in Jonesboro last summer, and recently with a stout negro man, in Atlanta, to the fullest extent, conjoined with taxis, without benefit. At least two gallons of water were used in the last case, and he thinks that the reduction might have been effected by it, and even with taxis alone, but for the protrusion of omentum, which was found to accompany the gut in its exit from the cavity through the internal abdominal ring. Thinks favorably of the theory, and that further trial may prove its usefulness for this purpose.

In a case of obstruction from supposed impacted cæcum, to which he was recently called, the good effects of distending and thoroughly sluicing the bowels with warm water were unmistakably manifested. A stout, young man of twenty-five years had suffered for two days with obstruction to the passage of fecal contents from the small intestines, with the usual distress and facial anxiety found in strictured bowels; also slight prominence and tenderness in the right iliac region. A few pints of water had been previously thrown into the rectum, but with the effect only of removing some contents of the colon. A gallon was then introduced, requiring considerable force, toward the close of the operation. This, with a small quantity of feces, was discharged in ten minutes—the patient being unable to retain it longer—without relief to the pain and other symptoms. An opiate was then given, and, after two hours rest, a gallon and a half of warm water was gradually thrown into the bowels, its escape during the introduction being prevented by a compress, through which the nozzle passed into the rectum. The discharge of this gave complete relief from suffering, and even before rising from the bed the patient felt comparatively easy. Up to the time of leaving, an hour and a half afterwards, there had been no return of pain. He infers that the patient was cured, or he would have been recalled.

Dr. Baird asked what Dr. Westmoreland meant by impacted cæcum.

Dr. W. replied, a cæcum impacted with feces or food, firmly jammed down in an impenetrable mass.

Dr. Battey suggested that surgeons had doubtless often felt the need, in obstinate cases of strangulated hernia, of a "manhole" in the side of the abdomen, through which a diminutive assistant could be sent inside to pull upon the incarcerated intestine whilst the taxis was exerted from without. This thought had several times suggested itself to his own mind. The needed force from within has been invoked in numerous instances, as reported in the journals, by the device of inverting the patient, the lower extremities being held upon the shoulders of an assistant, the body depending downwards, whilst the surgeon applied the external taxis, the gravity of the intestinal mass within dragging upon the incarcerated portion and greatly assisting the reduction. Those members who witnessed his experiments last year in the Academy, and saw the coil of intestine, twisted and knotted in a confused mass, so readily and rapidly untied and straightened out by simple distension with air, will be quite prepared to understand how valuable an aid similar distension with water must afford to the external taxis in the reduction of hernia. He called attention to the fact that water injected into the intestine, likewise, would enter the strangulated knuckle, and pass on through it, where the smallest channel of communication was left, and even soften, disintegrate, and wash out any contained mass of feces, thus diminishing the bulk of the hernia. He remarked also upon the action of the water as a dilator of the constricting ring. It is not necessary for the production of grave symptoms in strangulated hernia and intussusception that the caliber of the intestine shall be completely occluded. He has in his possession a specimen of fatal intussusception in which there were frequent liquid evacuations throughout the illness, which terminated fatally by exhaustion in three weeks time. At the autopsy the invagination, at the sigmoid flexure, permitted the easy passage of a cedar pencil through its caliber. In cases of serious intestinal obstruction he has uniformly observed a peculiar *facies*, which is not present in ordinary fecal impaction, and which he believes is entirely independent of any mental alarm, or, indeed, of any consciousness on the part of the patient of the serious character of the malady. He has observed this expression in very young children, who could not be supposed at all to realize the gravity of the situation; and this quite separate and distinct from any sense of pain experienced by the patient. He thinks he has observed the same *facies* in horses, and other animals, laboring under fatal intestinal obstruction. To develop fairly the value of the method in hernia and intussusception, it is necessary that the patient be anæsthetized; that the water be slowly thrown in, giving time for it to find its way onwards through its circuitous and tortuous route; that short intervals of rest be allowed, and the abdomen gently kneaded to encourage the upward passage of the liquid; and that the quantity of liquid shall be sufficient to fairly reach and fully distend the obstructed portion of intestine.

Dr. Conally had observation of a lady who had been closely observing *lent*, living upon crackers and tea chiefly. There was obstinatinate constipation, for which he directed simple purgatives and enemata, which were frequently repeated, and yet without satisfactory relief. Large distensile enemata brought away a large amount of feces, with complete relief.

Dr. W. F. Westmoreland had reported to the Academy some months ago a case of intestinal obstruction from grape seed in large quantity. The obstruction was removed by the distensile enema satisfactorily; but a troublesome enteritis followed, which ended fatally in five or six weeks.

Dr. Scott reported that he had charge of the case of varioloid reported heretofore by Dr. Conally. The patient is well, and no other case has occurred.

Dr. W. F. Westmoreland reported a case of anal fistula, wherein the external opening was at the base of the scrotum, while the internal opening was found upon the posterior wall of the rectum, above the sphinctre muscle. The tract of the fistula encircled the anus, and was very tortuous in its course. The operation required the following up of the sinus in several successive stages.

Dr. Conally remarked that he had a case some years ago of similar character to the one reported by Dr. W., in which he washed out the sinus, and injected it with a twenty grain solution of nitrate of silver. The patient passed from his observation, and he is unable to say whether a cure resulted.

Dr. Battey asked Dr. Westmoreland his experience in operating for fistula in case of tuberculous patients.

Dr. Westmoreland replied that he would fear that the fistula would not heal. If assured that it would heal he would still hesitate to operate.

Dr. J. G. Westmoreland asked if Dr. W. F. would operate hemorrhoids in phthisical subjects—to which he replied yes.

Dr. Battey thought there could be no valid objection to operating for the relief of fistula in cases of pulmonary tuberculosis, where a good healing of the fistula could be predicted. By removing this impediment to easy progression the patient may be turned loose in the woods with a shot-gun, and put in such sanitary conditions as would give good hope of a definitive stay of the pulmonary malady. In such case no harm, but on the contrary great good would be the result.

Dr. Calhoun remarked that he had recently operated six or eight cases of cataract in old persons ranging from seventy to seventy-six years of age. The books caution us that the aged bear the operation badly, because of the confinement to bed being irksome and damaging, and also that the corneal wound would heal with much greater difficulty. This has not been his experience in the cases alluded to. Sometime ago he operated an old man seventy-four years of age, who had paralysis agitans. He operated one eye with much difficulty and hazard. In the operation upon the other eye he used ether, and made the extraction with great ease. The paralysis passed off under the anæsthesia, and singularly it did not return for several days. Query: Is ether a hopeful remedy for paralysis agitans?

Dr. Baird remarked that tremor is merely clonic spasm of the muscles, which is relieved for the time by the ether, but returns when the influence of the ether is entirely gone.

Dr. Calhoun reported an observation yesterday of the case which he had trephined last April for epilepsy. For five months there was exemption from convulsions; since this he has had three attacks, but of a less severe character. For four months he took potassic bromide, but nothing since. Adjourned.

---

Atlanta, Ga., March 16, 1875.

Vice-President Taliaferro in the chair.

Dr. Armstrong reported case of fistula and hemorrhoids. The fistula was easily treated. The hemorrhoids were external, and were treated by simply splitting open the pile tumors one by one—with result of entire relief. There was no undue hemorrhage. He found a clot in the centre of each tumor—which was turned out with great relief to the patient at once—and a good cure.

Dr. Boring reported that the case of supposed gout, reported by him some months ago (see page 537 of last volume), is improving satisfactorily.

Dr. Conally reported case of dysmenorrhœa—lady of 30, with two children—very scanty means—has at each period a vicarious hemorrhage, which he believes comes from the throat, not from the lungs or stomach.

Adjourned.

---

ATLANTA, GA., March 23, 1875.

Vice-President Dr. V. H. Taliaferro in the chair.

Dr. J. G. Westmoreland reported case—man, aged 50—suffering but little pain—no appetite, eating scarcely anything—no fever, tongue red and slick, hard tumor in the epigastric region, towards the right side; not in the abdominal wall, but a tumor within the abdomen and located about the pyloric orifice. Took cathartic four or five days ago; acted freely; no evacuation for three days. Pain in abdomen, about the umbilicus, of a sharp, lancinating character. Vomited two days ago, but has had very little nausea. Has enjoyed excellent health until within three weeks. He asks the opinion of the Academy upon the diagnosis of the case. He suspects the possible existence of carcinoma.

Dr. Armstrong was of opinion that the case would likely prove to be carcinomatous. It is well known that the diagnosis in such cases, even at the bedside, was often obscure and difficult—certainly much more so here upon a recital of the symptoms merely.

Dr. Woodhull read a paper upon "*Clinical Studies, with the Non-Emetic Use of Ipecacuanha in Intermittent Fevers.*" Doses of one grain of the ipecacuanha in pill were given every four hours, without other medicine, and the results, as an antiperiodic, were essentially the same as obtained from the sulphate of quinine. The medicine seems to keep the bowels regular, but does not purge, nor does it vomit. In one case of enlarged spleen, doses of fifteen grains of bromide potassium, repeated three or four times daily, are rapidly reducing the enlargement.

Dr. Logan reported case of opium poisoning, seen in consultation with Dr. Roach—young man reported to have taken about 5½ ounces of laudanum three or four hours previously. Two or three emetic doses of sulphate zinc were exhibited without emesis. The stomach pump had been applied, but no fluid removed. Forty or fifty grains of caffein were exhibited with no improvement. Three doses of eighth grain of atropine were used hypodermically, with half hour intervals. He left the patient at 12

P.M., in what he regarded a hopeless state. Dr. Roach, who remained in attendance, repeated the atropine at 2 A.M. At 4 A.M. there was free vomiting, with odor of opium. The patient rallied and made a good recovery, having received in all a half grain of atropine. He regards the case as strongly confirmatory of the antagonism of atropine and morphine.

Dr. Calhoun had a case last summer, in which, by mistake of the druggist, he used hypodermically the tenth of a grain of atropine in place of strychnine. The patient was deeply poisoned by the atropine. He administered full doses of laudanum—one-half teaspoonful—every fifteen minutes, with prompt and marked improvement. The case exhibited markedly the antagonism of the two remedies.

Dr. Woodhull reported case of soldier who swallowed an ounce of laudanum last year. He used various remedies, but the hypodermic use of atropine was marked in its good effects.

Dr. W. F. Westmoreland had seen a paper recently in one of the journals detailing experiments which go to prove directly the contrary of the inferences drawn from the cases here reported.

Dr. J. T. Johnson had one case of opium poisoning in which the use of atropine did no good, and he feared had rather hastened the fatal result.

Adjourned.

---

ATLANTA, GA., March 30, 1875.

Vice-President Dr. V. H. Taliaferro in the Chair.

Dr. Taliaferro asked an opinion in cases of partial rupture of the perineum, extending only to the sphincter muscle, where there is no prolapsus of the uterus, nor loss of function of the spinctre—whether it is advisable to restore the perineum or not. In his case there is endometritis, and a sense of weakness of the perineum on defecation, which causes her instinctively to support the perineum with the hand.

Dr. Boring had but little experience in ruptured perineum, but thinks the discomfort of the patient would warrant an operation. He thinks care should be used to support the perineum in labor to guard against rupture. He is very careful to support the perineum well as the head passes over it, and has not had to encounter rupture in his practice.

Dr. Baird remarked that ruptured perineum occurred more often in the passage of the shoulder rather than the head. Pressure behind the anus about the point of the coccyx is useful, and more so than the mere support of the perineum solely.

Dr. Taliaferro had found in his cases that the shoulders and body passed with ease after the head is delivered, and thought the active agent in these lacerations of the perineum is the head of the child.

Dr. Battey said the object in view in the support of the perineum may be attained by placing two fingers on the head of the child, and thus retard its progress. The perineum is not touched, and the objection urged against perineal support is done away with. Moderate pressure will prevent any sudden and violent protrusion of the head. Called attention to another point. Cases in which the perineum is very rigid and the head of the child unduly large, the perineum may be protected by inserting two fingers into the anus and drawing it forward. He does not concur in the opinion that perineal support is pernicious. Is confident that lacerated perineum is an unnecessary bugbear to many members of the profession who, in their care to prevent it, even sacrifice the life of the child. He has encountered lacerated perineum on several occasions, usually in breach labors whilst earnestly engaged in rescuing the child. Such an occurrence does not mortify him. It sometimes occurs in the hands of the best obstetricians who are doing their whole duty. This fact ought to be recognized by the profession and known to the women of the land. Whilst he has been unfortunate in thus encountering ruptured perineum, he is happy upon the other hand that he has not been called upon to lament the loss of a child in labor, and is confident that he has rescued infants from death by disregarding the perineum in the weightier issue of the life of the child. He thinks the perineum, when ruptured, should be restored at once by sutures. The woman is then saved the shock to her delicacy which she feels so keenly at a later period, and the parts are so benumbed by the previous labor that the passage of the needle is but little felt. The happy mother does not weigh for a moment this little operation when offset against the life of her child. In one case of ruptured perineum in cephalic labor he distinctly observed, as is his custom, by passing the finger along the edge of the perineum, immediately after the escape of the head, that the structure was intact, whilst in the subsequent delivery of the trunk the perineum was cut by the elbow of the child in passing out.

He imagines the usefulness of pressure on the perineum is not, primarily, so much mere support of the perineum, but lies in pressing forward the passing part firmly under the pubic arch, keeping it in the line of Carus' curve, and preventing its passing off at a tangent. His experience concurs in the observation of Dr. Baird, that it is not the head of the child ordinarily, but the shoulder or elbow that causes the laceration. It is not uncommon, after the head has passed, that the arm flexed on the body causes the elbow to extend beyond the side, and it cuts the perineum in its passage. Is always careful to force in the elbow when it projects.

Attaches great importance to keeping obstetric patients in bed for ten days, and the use of the bed-pan. To this special precaution is due the infrequency of puerperal troubles in his practice. The day following the labor a bed-pan is placed under the patient, and a copious douche of warm water and castile soap is thrown into the vagina by means of a rubber bulb syringe. This is repeated twice daily, as long as the lochial discharge continues. He directs the labia to be slightly compressed around the pipe of the syringe, and the fluid so injected as to inflate the vagina. In this way the causes of metritis and septicæmia are in great part removed.

Dr. Taliaferro thought that it was a mistaken rule to confine patients any specified number of days. If the woman is healthy, and the reparative power is good, ten days is enough, but most women are weak, thin, muscular development is poor, and they should be kept in bed longer—for three weeks or a month. The cases that are in fine condition and stout are rare. His own opinion is that the very large number of uterine diseases that now flood the country are, in many instances at least, caused by getting up too early. The uterus is large and heavy; its supports are lax; the muscles are lax; the perineum is sometimes ruptured. Subinvolution and its long train of evils is the result.

Dr. Battey replied to queries propounded, that he regarded the obstetrical binder, when properly applied, as of use in supporting the abdominal contents and maintaining good contraction of the uterus, as well as conducing to the comfort of the *acouche;* and, moreover, especially useful in preserving the tone of the abdominal walls and smooth, regular contour of the abdomen. He instanced the case of a lady who had been repeatedly under his care in the lying-in chamber; a lady of middle age, the mother of thirteen children, who had uniformly worn a broad and well-fitting binder for thirty days following each confinement, whose abdomen to-day is as well supported, as smooth and as handsomely rounded as it was upon the day of her marriage. He thought this result attributable to the careful use of the binder. He did not approve of the practice recommended by Dr. Goodel of the Preston Retreat, Philadelphia, of the patient "slipping out of bed into a chair" on the second or third day after labor, and daily thereafter. He attached great importance to the horizontal position, to be carefully maintained for at least ten days, with the use of the bed-pan for evacuations. So far as the escape of offensive lochia by gravitation, to be secured by the erect posture, is concerned, he thought this was much better attained by a thorough washing out of the vagina, morning and evening, with warm soap suds, by means of a suitable elastic syringe, over the bed-pan, a much better practice, and should be continued daily as long as the lochia lasted. Careful attention to this removes the puerperal odor of the lying-in chamber, and aids much in protecting the patient against puerperal metritis and septic poisoning.

He had not felt the need of Crede's method of expelling the placenta after labor, nor did he esteem the rude compression of the uterus, to force out the placenta into the vagina, either necessary or proper. It is his practice not to wait for the spontaneous expulsion of the placenta, but, having secured good uterine contraction, by gently rubbing the abdomen over the globe, by a cold, wet hand, by a few drops of cold water suddenly applied, or other suitable means, he proceeds at once to complete the delivery, by making gentle traction upon the cord with the left hand, whilst two fingers of the right are introduced to hook down the edge of the placenta, which is seized between the fingers and rotated so as to turn the placenta in the uterus, and thus excite expulsatory contractions, which, aided by gentle tension upon the cord, he has found uniformly effective and satisfactory in all cases where the placenta is thrown off from its uterine attachment. If we have an adherent placenta, he does not think that Crede's method can be trusted to effect a clear and proper detachment of the adhesions. For this purpose he deems it a safer and better practice to introduce the hand into the uterus, and, spreading the fingers, to seize the placenta by its periphery, rotating it upon its axis in such a manner as to detach it cleanly and entire from the uterine wall. He attaches importance to the subsequent removal of all clots from the uterus and vagina. We thus avoid distressing after pains, and secure more permanent contraction of the uterus and exemption from post-partum hemorrhage. Adjourned.

---

### AN ESSAY ON THE ACTIONS OF MERCURY.

By J. G. WESTMORELAND, M.D.

*Read before the Academy of Medicine February* 9, 1875.

The action of remedies is defined in two different and entirely distinct aspects, denominated physiological and therapeutic. Progress in medicine, however, is gradually but surely leading to the adoption of the more practical idea, that therapeutic changes are only the results of, and not the actions themselves. Much confusion is found, not only with the student of medicine, but advanced members of the profession, by the loose manner in which the action of medicines is alluded to, in works on therapeutics, essays, reports in practical medicine, etc.

By the action of remedies we must be understood to mean the physiological alterations effected in parts without regard to the existence of disease at all. Lesions of structures and functional changes may be made by this action, and are produced in health as well as disease. It is called physiological action by the great American and European acologists, Wood and Pereira, and is induced in order to therapeutic results, in the alterations brought about in diseased parts by such action, and the therapeutic changes are called direct or indirect, accordingly as the action is exerted on the diseased or a sound part. For example, when sulph. copper or nit. silver is applied to an ulcer, the therapeutic result is called direct, but the action of a blister upon the skin, and of veratrum upon the heart, in the relief of pleuritis, the therapeutic change is said to be the indirect result of the action of these remedies. In addition to what has already been said touching the definition of action of remedies, I should mention the fact that certain remedies are called local, having action upon the part to which they are directly applied: some elective, general or constitutional, acting upon parts after having entered the circulation and carried to the organ or tissue of their election; while others act both locally and electively, affecting the tissue to which they are applied, and, after being absorbed into the circulation, impress other parts also, and perhaps several at the same time. A familiarity with the physiological action of remedies enables the practitioner to use them rationally and successfully in the treatment of disease, and without which prescriptions are made by routine and empirically.

All the preparations of mercury have, uniformly, the same elective or general actions, when taken into the circulation, but some differ from others very materially in their local action. While the mild chloride and blue mass have no perceptible impression when applied locally, except perhaps the absorbent, drying property of the former, yet the corrosive chloride and nitrate are excessively irritating to all soft structures. These last are soluble preparations, and, therefore, would seem to be more appropriately used for the introduction of mercury into the circulation, but, on account of the local irritation produced by them in the stomach or other parts to which they are applied for absorption, the former preparations, though insoluble in watery fluids, are preferred in most instances when the remedy is used for its elective actions.

The properties of this metal are various and important. So much so that routinists rarely expect criticism, when they use some of its preparations in every disease that presents itself. Under the term "alterative," it would seem to be universally applicable to every pathological condition that ever has or ever can occur. This term, which originated in empiricism, answers to cover ignorance of pathology and the true physiological action of the remedy. It was ascertained that lost health was restored by mercury under certain circumstances, and, without inquiry into the pathology or *modus operandi* of the remedy in the cure, the action was named with reference to the alteration or change from disease to health. A little more definite, but not sufficiently so for the present advancement in medicine, is the name having reference to the therapeutic result obtained by its use in particular diseases, such as antisyphilitic action, antiphlogistic action, etc.

Mercury, when taken into the circulation, affects certain organs, neutralizes certain morbific agents in the blood and in solid structures, and destroys certain natural ingredients of the blood itself. Also, when placed in contact with any known parasite of the human body, upon which experiments have been made, destructive action is exerted upon the worm or insect.

Various and contradictory views are entertained in regard to the action of mercury on internal organs. While some believe the whole secernent system is excited to increased secretion, others feel equally certain that few, if any, of the glands are affected by the remedy. A happy medium between these extreme views is perhaps safer ground to occupy. That the liver and salivary glands are impressed, and the latter sometimes to a degree of inflammatory excitement, there seems to be abundant proof. The excessive flow of saliva, and tenderness of the salivary glands and gums, from the elective action of absorbed mercury, has been too often witnessed to admit of reasonable doubt. Not so positive are the evidences of hepatic stimulation, but sufficiently so, it is believed, to warrant its administration for this purpose. The result of carefully-made statistical experiments upon lower animals, however, has been strongly urged against this opinion. It was ascertained that mercury, given to a dog with normal condition of the liver, did not increase the amount of bile, and upon this fact the accepted doctrine of its hepatic stimulation is denied. We do not think the conclusion is legitimate. An organ in a normal condition, whose function is healthfully performed, may be stimulated to the extent of entirely suspending its function. Who does not know that renal stimulants may excite the kidneys to the extent of materially impairing their function? When the kidneys are in a normal condition, turpentine and other excitants of these organs, in large doses, lessen the amount of urine secreted, while the same remedies will increase the quantity, if given when the kidneys fail to perform their function, from a want of sufficient excitement. Organs fail to perform their functions properly, on account of over stimulation, as well as from deficiency; and, as in the experiments alluded to, when a healthy organ is stimulated, the function may be less perfectly performed. Had the liver been inactive, and the bile deficient from want of excitement, when the mercury was given to the dog, the amount would, in all probability, have been increased.

In hepatitis, when the inflammatory excitement lessens the secretion of bile, mercury could not be expected to increase it; but, in the opposite condition, when no evidence of bile is seen in the stools, the use of this remedy is followed by unmistakable signs of its presence in the alimentary canal. It is in such *non-bilious* or *anti-bilious* condition, depending upon inactivity of the liver, and this only, that mercury will prove effectual in restoring the secretion of proper amount of this fluid. The use, therefore, of its preparations as a stimulant to the liver, in a bilious condition of the system, is unnecessary and sometimes injurious. This state is characterized by icterous hue of the skin and eyes, and is often the result of obstruction to the passage of bile into the duodenum, from congestion and inflammation of the gut, and its consequent absorption and deposit in these parts, as in ordinary jaundice. Sometimes obstruction in the small intestines, below the entrance of the bile duct, by impacted fæces, may lead not only to absorption to some extent, but to bilious vomiting from regurgitation into the stomach. This is often the case in autumnal fever, giving rise, perhaps, to the term "bilious fever," applied to it. Under such circumstances, the cathartic effect of mercury may open up a channel for the passage of bile through the bowels, independent of any elective influence upon the liver. Catharsis, from the action of any remedy, may give, in a similar manner, the evidences of bile in the alvine evacuations where they had been absent before.

This action of mercury upon the bowels seems to be not only independent of its elective stimulation to the liver, but more of a local influence than otherwise. Its presence in the canal appears to be necessary for catharsis, while absorption into the circulation is essential to its elective action upon the liver and other parts. The arrest of catharsis, and of the consequent passage of the mercurial through the canal, favors the absorption and its elective or constitutional actions, and, *vice versa*, the encouragement of alvine discharges prevents these in a good degree. In order to insure hepatic stimulation, therefore, it is necessary to give opium, or some other constipating remedy, in connection with the mercurial. When the ointment of mercury is applied to the skin, and taken directly into the circulation by absorption from this surface, no tendency to catharsis is had by it, while all the elective effects will be manifested.

Mercury, as a catalytic, is extensively and profitably used. It is given in large doses for the destruction of fibrine in the blood, when active antiphlogistic treatment becomes necessary in acute inflammation. The soothing, sedative effect of a full quantity of the remedy is also manifested under such circumstances, and in the control of convulsive and other nervous disturbance from direct impression upon the centres, by sudden cessation after constant use of alcohol, or from reflex nervous derangement, by irritation of worms, etc.

As a solvent and eliminator of syphilitic virus, the catalytic action of mercury is more distinctly marked. Its administration is considered almost indispensable in the treatment of this constitutional contamination. For more than a century past it has been considered the great syphilitic "alterative." In ridding the system of this contagious morbific agent, its action was, for a long time, and is even now, by some, looked upon as mysterious and unaccountable. Is the result obtained subject to any rational explanation other than that of direct destruction of the virus by solution and elimination? I think not. The theory of restoring health, under these circumstances, by giving increased vigor to the assimilative organs and promoting nutrition, is not at all satisfactory to my mind. The name eutrophic, given to this class of remedies by Prof. Dunglison, under the impression that nutrition is promoted, shows a disposition to locate the action somewhere; but it would, perhaps, be better to retain the term "alterative," whereby we acknowledge our ignorance of the action had, rather than change it for a name indicating an action not produced at all. However perfect the nutrition may be, syphilitic virus, if left in the system, will prove destructive to health by the development of local lesions in various parts of the body. It is not, therefore, by any action exerted upon the natural tissues or organs of the body that syphilis is cured, but by solution and elimination of the virus.

When the preparations of mercury are given for enteric irritation and inflammation, less than cathartic doses are used, with the view of exciting the torpid liver to action, and thereby give free transmission of blood through the organ from the bowels, which had become congested and inflamed on account of sluggish portal circulation. This is a very rational course of procedure; but there is no doubt that sometimes the enteric inflammation is aggravated by small doses of calomel frequently repeated. Enough free hydrochloric acid is often found in the stomach to convert small quantities of mild chloride into the corrosive, and thus form a local irritant calculated to increase excitement in the alimentary mucous membrane. The old custom of giving repeatedly small doses of calomel, in the treatment of cholera infantum, as recommended by Dewees and others, is objectionable, to my mind, on this account. Large doses, affording more mercury than is readily changed to the corrosive salt, would, therefore, seem to be more appropriate; and those who have witnessed the persistent diarrhœal discharges and vomiting, in children treated with frequent small doses of calomel for several days, will, I think, testify of this.

